# The adjuvants dmLT and mmCT enhance humoral immune responses to a pneumococcal conjugate vaccine after both parenteral or mucosal immunization of neonatal mice

**DOI:** 10.3389/fimmu.2022.1078904

**Published:** 2023-01-20

**Authors:** Jenny Lorena Molina Estupiñan, Audur Anna Aradottir Pind, Poorya Foroutan Pajoohian, Ingileif Jonsdottir, Stefania P. Bjarnarson

**Affiliations:** ^1^ Department of Immunology, Landspitali, The National University Hospital of Iceland, Reykjavik, Iceland; ^2^ Faculty of Medicine, School of Health Sciences, University of Iceland, Reykjavik, Iceland

**Keywords:** vaccination, neonates, adjuvants, mucosal immunization, antibodies, germinal center, antibody-secreting cells (ASC)

## Abstract

Immaturity of the neonatal immune system contributes to increased susceptibility to infectious diseases and poor vaccine responses. Therefore, better strategies for early life vaccination are needed. Adjuvants can enhance the magnitude and duration of immune responses. In this study we assessed the effects of the adjuvants dmLT and mmCT and different immunization routes, subcutaneous (s.c.) and intranasal (i.n.), on neonatal immune response to a pneumococcal conjugate vaccine Pn1-CRM_197_. Pn1-specific antibody (Ab) levels of neonatal mice immunized with Pn1-CRM197 alone were low. The adjuvants enhanced IgG Ab responses up to 8 weeks after immunization, more after s.c. than i.n. immunization. On the contrary, i.n. immunization with either adjuvant enhanced serum and salivary IgA levels more than s.c. immunization. In addition, both dmLT and mmCT enhanced germinal center formation and accordingly, dmLT and mmCT enhanced the induction and persistence of Pn1-specific IgG^+^ Ab-secreting cells (ASCs) in spleen and bone marrow (BM), irrespective of the immunization route. Furthermore, i.n. immunization enhanced Pn1-specific IgA^+^ ASCs in BM more than s.c. immunizatiofimmu.2022.1078904n. However, a higher i.n. dose of the Pn1-CRM_197_ was needed to achieve IgG response comparable to that elicited by s.c. immunization with either adjuvant. We conclude that dmLT and mmCT enhance both induction and persistence of the neonatal immune response to the vaccine Pn1-CRM_197_, following mucosal or parenteral immunization. This indicates that dmLT and mmCT are promising adjuvants for developing safe and effective early life vaccination strategies.

## 1 Introduction

Vaccination is one of the most cost-effective ways to prevent a variety of infectious diseases, and early life vaccination has reduced deaths in neonates and children across the world (1). Although substantial progress has been made in recent decades, infectious diseases are still one of the leading causes of death of children under five years of age (2). Vaccination in the neonatal period has its challenges due to immaturity of the immune system leading to increased susceptibility to infectious diseases.

Neonatal immunizations induce low antibody (Ab) responses and multiple vaccinations are needed to maintain protective immunity and immunological memory (3). Multiple factors contribute to poor Ab response in infancy, including: 1) decreased expression of co-stimulatory molecules which result in limited cross-talk between dendritic cells (DCs), T cells and B cells (4), 2) dampened B cell germinal center (GC) reaction due to poorly developed follicular dendritic cells (FDC) (5), 3) preferential differentiation of memory B cells rather than plasmablasts (6), 4) reduced survival of long-lived plasma cells in the bone marrow (BM) due to insufficient survival signals (7, 8).

Intranasal (i.n.) immunization elicits antigen-specific immune responses in the respiratory tract (9). Mucosal immunization of experimental animals with polysaccharide-protein conjugate vaccines against encapsulated respiratory bacteria, like pneumococcus, meningococcus and *H. influenzae*, induces both mucosal and systemic immune responses, providing protection against carriage, otitis media and invasive disease in a variety of challenge models (10). Conjugate vaccines are highly efficacious against invasive diseases, but their efficacy against carriage and mucosal infections might be improved by i.n. immunization (11, 12). Importantly, genesis of the nasal associated lymphoid tissue (NALT) begins early after birth and is thought to be triggered by stimulatory signals provided by environmental antigens and mitogens (13). This is supported by results showing that nasal administration of cholera toxin, a well-known mucosal antigen and adjuvant, resulted in accelerated organogenesis of NALT and development of the bell shaped lymphoid tissue (14). Therefore, i.n. immunization is an attractive approach for early life immunization against respiratory pathogens including encapsulated bacteria. I.n. immunization requires safe and effective adjuvants to increase vaccine uptake and induce antigen-specific protective immunity although other novel nasal vaccine delivery systems are also being developed (15).

Adjuvants can stimulate and enhance the magnitude and duration of the immune response (16–18). Currently, the adjuvant alum is licensed in multiple human infant vaccines and MF-59 is licensed for H1N1 influenza vaccination of infants from 6 months of age (19–21). Various novel adjuvants are being assessed in pre-clinical neonatal models and some have been shown to overcome limitations of early life immunity (11, 22–26). We have shown that the adjuvants LT-K63, MF-59, mmCT and IC31 given subcutaneously (s.c.) with a pneumococcal conjugate vaccine (Pnc1-TT) to neonatal mice enhanced GC induction and elicited higher and sustained Ab responses, sufficient for protection against pneumococcal bacteremia and lung infection (25). Using a well-established murine model of immunization and infection we have shown that in neonatal, infant and adult mice, Pn1-specific IgG Ab level of log 1.5 EU/ml and log 2.5 EU/ml is protective against pneumococcal bacteremia and lung infection, respectively, when mice are challenged i.n. with a high dose of *S. pneumoniae* of serotype 1 (11, 12, 27–29). Using the same model, we showed that LT-K63 is a potent mucosal adjuvant when given with Pnc1-TT to neonatal mice, inducing protective immunity against lethal pneumococcal infections, where i.n. immunization was superior to s.c. immunization in the induction of both primary and memory responses and protective capacity, especially after only one immunization (11, 12).

In this study, we assessed the effects of two adjuvants, dmLT and mmCT, on neonatal immune responses and compared i.n. and s.c. routes of immunization with a pneumococcal conjugate vaccine, Pn1-CRM_197_. dmLT is a double mutant detoxified version of the heat-labile enterotoxin of *Escherichia coli* (30), and mmCT is a non-toxic multiple mutant of cholera toxin (CT) (31). CT and LT are the most potent mucosal adjuvants known today but cannot be used in human vaccines due to their toxicity. Enzymatically inactive mutants of CT and LT, such as mmCT and dmLT, have been shown to still possess adjuvant effects while exhibiting low or no toxicity. In adult mice both adjuvants increased serum IgG, mucosal IgA responses and CD4^+^ T cell responses, especially Th17 cells (32, 33). dmLT improved immune responses following oral, sublingual, intradermal and intramuscular immunization (34–37). Furthermore, dmLT has shown good safety profile and strong adjuvanticity in clinical trials with an oral vaccine for enterotoxigenic *E. coli* (ETEC) (38–40). In this study we demonstrate that dmLT and mmCT can overcome limitations of the early life immune system where they enhance both induction and persistence of humoral immune responses when administered either s.c. or i.n. However, the vaccine dose had to be increased when given i.n. to elicit comparable protective IgG Ab levels as after parenteral immunization. Furthermore, the mucosal route had the benefits of enhanced mucosal and systemic IgA responses.

## 2 Materials and methods

### 2.1 Mice

Adult NMRI mice (5-6 weeks old) were purchased from Taconic (Skensved, Denmark). They were kept in microisolator cages with free access to commercial food pellets and water and housed under standardized conditions at ArcticLAS vivarium facility (Reykjavík, Iceland), with regulated temperature, daylight and humidity. Breeding cages were checked daily for new births, and pups were kept with their mothers until weaning at 4 weeks of age. The protocol was approved by the Experimental Animal Committee of Iceland, according to regulations 279/2002.

### 2.2 Antigen, adjuvants and immunization

The pneumococcal conjugate vaccine Pn1-CRM_197_ was provided by the Serum Institute of India (India) and consisted of a pneumococcal polysaccharide of serotype 1 (Pn1) conjugated to diphtheria toxoid (CRM_197_) (41). The adjuvants mmCT and dmLT were produced as described previously (30, 31) and provided by the Vaccine Research Institute (University of Gothenburg, Sweden).

To determine the optimal dose of the pneumococcal conjugate vaccine Pn1-CRM_197_, neonatal (7 day old) mice (8 mice per group) were immunized once s.c. at either side at base of tail with 0.5, 0.75 or 1 µg of Pn1-CRM_197_ and Pn1-specific IgG Abs were measured bi-weekly up to 8 weeks. Based on the results (**Supplementary Figure 1**), 0.75 µg per dose was selected for experiments testing the effect of mmCT and dmLT on neonatal immune response to s.c. and i.n. immunization with Pn1-CRM_197_.

Neonatal mice were immunized with 0.75 µg of Pn1-CRM_197_ with or without 2 or 5 µg of dmLT or mmCT. To test the effects of increased vaccine doses for i.n. immunization neonatal mice were immunized with 0.75, 2 and 4 µg of Pn1-CRM_197_ with or without 5 µg of dmLT or mmCT. The vaccine solutions were mixed 1 h prior to immunization. For i.n. immunization two doses of 2.5 µL of vaccine solution were slowly delivered into the nares, with 30 min between doses. Anesthesia was avoided to limit aspiration into the lungs. For s.c. immunization 50 µL of vaccine solution was injected at the base of tail.

### 2.3 Blood and saliva sampling

Mice were bled from the tail vein bi-weekly up to 8 weeks after immunization for measurement of anti-Pn1 Abs in serum. Saliva was collected from each mouse by the insertion of absorbent sticks (Polyfiltronics Inc., Rockland, Maine) into the mouth. After 5 min, the sticks were transferred to phosphate-buffered saline (PBS) containing 10.0 µg of protease inhibitor (aprotinin; Sigma-Aldrich, St Louis, MO, USA) per ml to prevent proteolysis. The dissolved saliva samples for each group were pooled and stored at -70°C.

### 2.4 Enzyme-linked immunosorbent assay

Pn1-specific serum and salivary Abs (IgG and IgA) were measured by ELISA as described previously (29). Briefly, microtiter plates (MaxiSorp; Nunc AS, Roskilde, Denmark) were coated with 5 μg/µL of Pn1 (American Type Culture Collection, Rockville, MD) in PBS for 5 h at 37°C. After that, the plates were washed and blocked with PBS with 0.05% Tween 20 containing 1% BSA (Sigma). Serum samples and standard were neutralized with cell wall polysaccharide (CWPS; Statens Serum Institute), diluted 1:50 (or 1:25, if needed) in PBS with 0.05% Tween 20 (Sigma) and incubated in 500 μg/mL of CWPS for 30 min at room temperature. Neutralized serum samples and standard were serially diluted whereas salivary samples were left undiluted and both were incubated for 2 h in duplicates in Pn1-coated microtiter plates at room temperature. The plates were washed and then incubated with horseradish peroxidase conjugated goat anti-mouse IgG or IgA (Southern Biotechnology Associates, Birmingham, AL) diluted 1:5,000 in PBS-Tween for 2 h at room temperature. The plates were washed again and 3,3′,5,5′-tetramethylbenzidine-substrate (Kirkegaard & Perry Laboratories, Gaithersburg, MD) was used for development and the reaction stopped with 0.18 M H2SO4. Absorbance was measured at an optical density of 450 nm in Multiskan FC Microplate Photometer (Thermo scientific, Waltham, Massachusetts, USA). The standard for Pn1-specific Abs was obtained by isolating sera from adult mice hyper-immunized with the conjugate vaccine. The results were calculated from a standard curve and expressed as mean log of ELISA units (EU)/ml for IgG and mean of EU/ml for IgA.

### 2.5 Enzyme-linked immunospot

Pn1-specific IgG^+^ and IgA^+^ ASCs were enumerated by ELISpot, as previously described (22), in spleen and BM 14 and 56 days after immunization. MultiScreen High protein binding immobilon-P membrane plates (Millipore Corporation, Bedford, MA) were coated with 10 µg/ml Pn1 overnight at 37°C, blocked with complete RPMI 1640 (Life Technologies BRL, Life Technologies, Paisley, U.K.). Duplicates of cells from spleen and BM were incubated in four threefold dilutions starting with 1 × 10^7^ cells in 100 µL of complete RPMI 1640 per well for 5 h at 37°C, washed and incubated with ALP-goat anti mouse IgG or IgA (Southern Biotechnology Associates) overnight at 4°C, and developed by 5-bromo-4-chloro-3-indolylphosphate and NBT in AP development buffer (Bio-Rad Labs, Hercules, CA). The number of spots (each representing a cell secreting specific Abs) were counted by ELISPOT reader ImmunoSpot R S6 ULTIMATE using ImmunoSpot R SOFTWARE (Cellular Technology Limited (CTL) Europe, Bonn, Germany).

### 2.6 Immunohistohemistry

GC formation was studied by staining spleen sections with peanut agglutinin (PNA) and anti-IgM, as described elsewhere (22). Spleens collected 2 weeks after immunization were frozen using Tissue-Tek OCT (Sakura, Zouterwoude, the Netherlands) and cut into 7 µm cryosections at 2 levels, starting 1,750 µm into the tissue; the levels were separated by 210 µm, fixed in acetone for 10 min, and stored at −70°C. One section from each level for each mouse was stained with fluorescent labeled IgM-FITC (BD Pharmingen) to visualize the follicles, and biotinylated PNA-bio (Vector Laboratories, Burlingame, CA) to label GC B cells, and incubated at room temperature for 30 min. The sections were then washed in PBS for 2 × 5 min prior to incubation with APC Streptavidin (BD Pharmingen) at RT for another 30 min and sections washed again as before. DAPI (Invitrogen, Eugene, OR) was used for nuclear counterstaining. The sections were photographed with a digital camera (AXIOCAM; Zeiss) in a microscope (Zeiss) equipped with x10 and x40 objectives and Zenblue Software (Birkerod, Denmark) for light and three-color immunofluorescence. Areas of PNA^+^ staining were measured from all pictures using the Zenblue Software.

### 2.7 Statistical analysis

Statistical analyses between groups were performed using the Mann Whitney U test or Kruskal Wallis H test (*p ≤ 0.05, **p ≤ 0.01, ***p ≤ 0.001) using Graphpad Prism 6.0 (GraphPad Software, La Jolla, CA). P-values less than 0.05 were considered statistically significant.

## 3 Results

### 3.1 The adjuvants dmLT and mmCT enhance vaccine-specific IgG response both after intranasal and subcutaneous immunization of neonates

We have previously shown that mucosal immunization with a pneumococcal conjugate vaccine, Pnc1-TT, and the adjuvant LT-K63 enhanced protective immunity against lethal pneumococcal infections in neonatal mice, where a single i.n. dose of Pnc1-TT with LT-K63 induced significantly higher protective IgG responses than s.c. immunization (11). To evaluate the potential of i.n. immunization with the potent mucosal adjuvants dmLT and mmCT neonatal mice were immunized once i.n. or s.c. with 0.75 µg of Pn1-CRM_197_ with or without dmLT or mmCT or injected with saline as a control. Since a different pneumococcal conjugate, Pn1-CRM_197_, was used in this study a pilot experiment was performed where several doses of Pn1-CRM_197_ s.c. were tested and 0.75 µg was found to be optimal (**Supplementary Figure 1**). We additionally compared the effects of two different doses of either adjuvant, 2 µg and 5 µg. We have reported that 2 µg of mmCT enhances both IgG Abs and IgG^+^ ASCs in our neonatal mouse model, when given s.c. with Pnc1-TT (25) and TT (42). In adult mice, mmCT and dmLT at 1.5 to 10 µg doses have been shown to increase immune response to different antigens administered by mucosal or parenteral immunization routes (31, 37, 43, 44).

**Figure 1:**
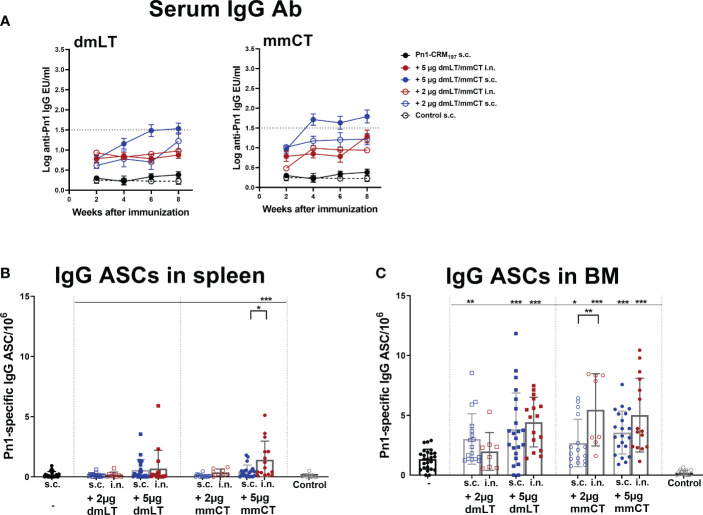
Adjuvants dmLT and mmCT enhance humoral immune responses irrespective of the immunization route. Pn1-specific serum IgG Ab levels 2, 4, 6 and 8 weeks **(A)** and number of Pn1-specific IgG^+^ ASCs in spleen **(B)** and BM **(C)** 8 weeks after s.c. or i.n. immunization of neonatal mice with Pn1-CRM_197_ with or without 2µg or 5µg of adjuvants dmLT or mmCT or saline-injected mice as controls **(A)**. Results are expressed as IgG levels (log mean EU/ml ± SEM), and number of spots/10^6^ cells (mean ± SEM), in 3 comparable independent experiments combined (except the groups immunized i.n. with Pn1-CRM_197_ with 2µg of dmLT or mmCT where there are 8 mice per group). Statistical difference was calculated using Mann–Whitney U-test where adjuvant groups were compared to vaccine only group and immunization routes were compared between the adjuvanted groups. *p ≤ 0.05, **p ≤ 0.01, ***p ≤ 0.001. The dotted line representes protective IgG Ab levels for pneumococcal bacteremia.

Pn1-specific IgG Ab levels in serum of neonatal mice immunized s.c. with Pn1-CRM_197_ alone were low and comparable to those of saline-injected mice. However, inclusion of either adjuvant enhanced Ab responses, irrespective of the immunization route (**Figure 1A** and **Supplementary Table 1**). Furthermore, the 5 µg dose of dmLT or mmCT was more effective than 2 µg in enhancing IgG Ab levels since Pn1-CRM_197_ immunization with 5 µg of either adjuvant induced higher Ab levels at all time points (p ≤ 0.001) than Pn1-CRM_197_ alone (**Supplementary Table 1**). Of note, i.n. immunization with Pn1-CRM_197_ and 5 µg of dmLT or mmCT elicited lower IgG Ab levels than s.c. immunization overall (Kruskal Wallis, p ≤ 0.001), where the difference becomes pronounced after week 4. Accordingly, 54% and 76% of neonatal mice immunized s.c. with Pn1-CRM_197_ and 5 µg dmLT or mmCT respectively reached Pn1-specific IgG levels known to be protective against pneumococcal bacteremia (11, 27), whereas with i.n. immunization, 0% of the mice that recieved dmLT and 33% of those that recieved mmCT reached protective levels against bacteremia.

We have demonstrated that several adjuvants can enhance the induction of vaccine-specific ASCs and prolong their persistence in the BM in neonatal mice (22, 25, 26, 42). Therefore, we wanted to assess if mmCT and dmLT had similar effects and whether immunization routes affected these parameters differently. To do so we measured IgG^+^ ASCs in spleen and BM 8 weeks after i.n. or s.c. immunization (**Figures 1B, C**). The inclusion of dmLT and mmCT, especially the 5 µg dose, at either immunization route enhanced the number of IgG^+^ ASCs in the BM compared to immunization with Pn1-CRM_197_ alone (**Figure 1C**). Furthermore, only mice immunized i.n. with 5 µg of mmCT showed persistence of ASC in spleen upto this late timepoint, as they had higher number of Pn1-specific ASC than mice immunized with the same vaccine formulation by the s.c. route or with the vaccine alone (**Figure 1B**). These results demonstrate that both mucosal and systemic immunization with either mmCT or dmLT increase the persistence of specific IgG^+^ ASCs in BM to a similar degree even though systemic immunization seems to be more effective in enhancing Pn1-specific IgG serum Abs.

### 3.2 dmLT and mmCT enhance both mucosal and systemic vaccine-specific IgA responses after intranasal immunization of neonates

**Figure 2:**
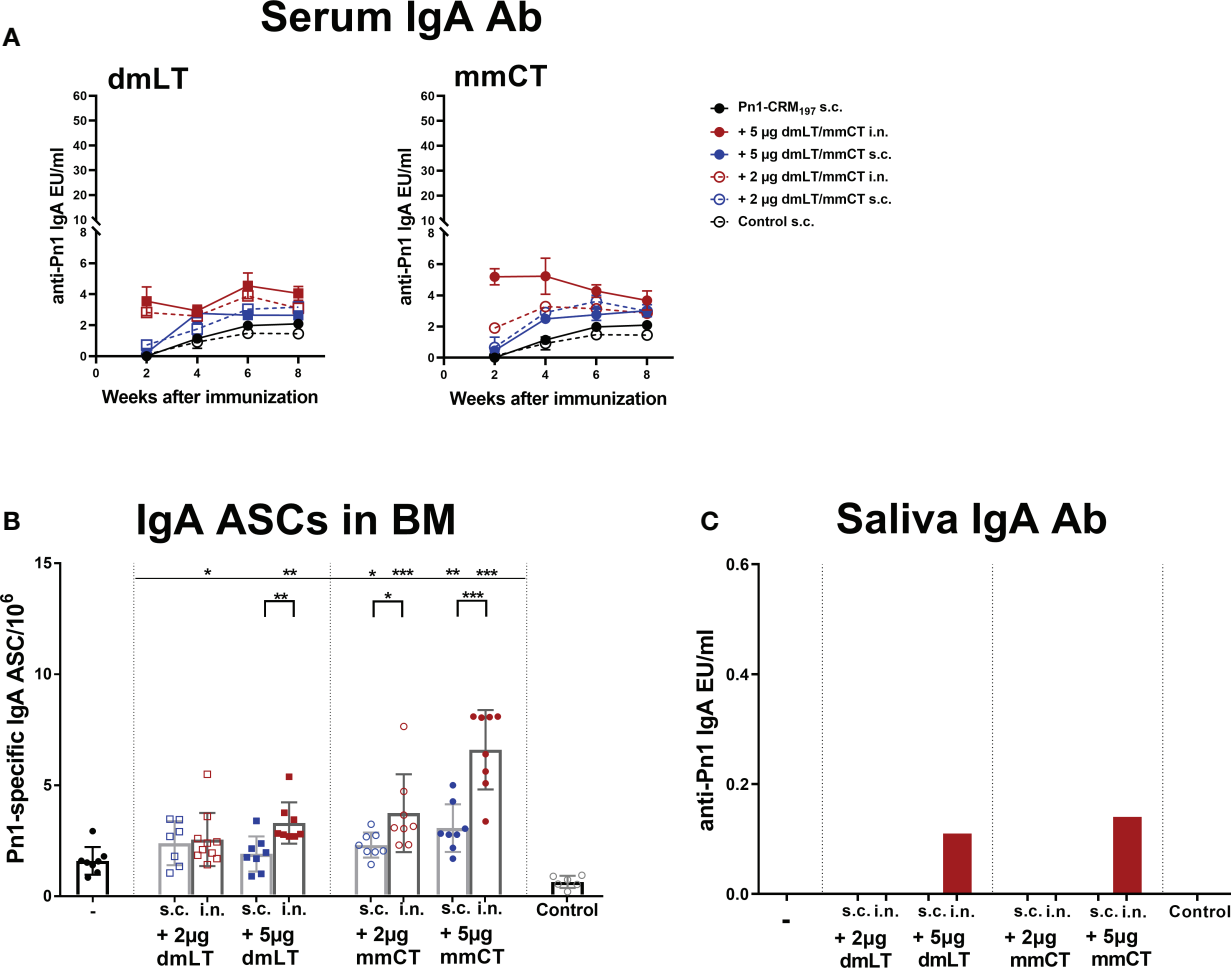
Intranasal immunization of neonates with adjuvants dmLT and mmCT enhance both systemic and mucosal IgA responses. Pn1-specific serum IgA Ab levels 2, 4, 6 and 8 weeks **(A)** and number of of Pn1-specific IgA^+^ AbSCs in bone marrow **(B)** 9 weeks after s.c. or i.n. immunization of neonatal mice with Pn1-CRM_197_ with or without 2µg or 5µg of adjuvants dmLT or mmCT. Results are expressed as IgA levels (EU/ml ± SEM) in 3 comparable independent experiments combined and as number of spots/10^6^ cells (mean ± SD) in 7–8 mice per group. Saliva was collected 8 weeks after immunization and Pn1-specific IgA Abs were measured in saliva samples by ELISA **(C)**. Statistical difference was calculated using Mann–Whitney U-test where adjuvant groups were compared to vaccine only group and immunization routes were compared between the adjuvanted groups. *p ≤ 0.05, **p ≤ 0.01, ***p ≤ 0.001.

Mucosal immunization induces humoral and cell-mediated immune responses in both mucosal and systemic compartments (10). Therefore, we assessed the effects of dmLT and mmCT (2 and 5 µg) and the effect of the immunization route on Pn1-specific IgA serum levels and IgA^+^ ASCs. Serum IgA levels were overall low in mice immunized s.c. with Pn1-CRM_197_ alone. I.n. immunization with Pn1-CRM_197_ and 5 µg of dmLT or mmCT enhanced serum IgA Abs compared to Pn1-CRM_197_ s.c. alone at all time points (p ≤ 0.001), whereas 2 µg of the adjuvants had less effect, similar to what was observed for IgG responses. The effects of the adjuvants on IgA levels were most pronounced at early time points (**Figure 2A** and **Supplementary Tables 3, 4**). On the contrary, s.c. immunization with Pn1-CRM_197_ and 5µg of dmLT or mmCT yielded a slight increase in IgA levels but they were generally lower than after i.n. immunization (**Figure 2A** and **Supplementary Table 3**). These results indicate that i.n. immunization of neonates with dmLT or mmCT with Pn1-CRM_197_ can rapidly enhance the induction and persistence of Pn1-specific serum IgA Abs. Accordingly, both dmLT and mmCT enhanced the number of Pn1-specific IgA^+^ ASCs in BM (**Figure 2B**). Thus, i.n. immunization with dmLT (5 µg) or mmCT (5 or 2 µg) induced higher numbers of IgA^+^ ASCs in the BM than s.c. immunization with the corresponding vaccine formulation.

Since mucosal IgA can protect against colonization of pneumococci (29) and potentially reduce invasion and disease (9, 10, 45), we also measured Pn1-specific IgA in saliva, where samples were pooled for each group. Mice immunized i.n. with Pn1-CRM_197_ and 5 μg of dmLT or mmCT had high salivary IgA levels, whereas in s.c. immunized mice and mice immunized i.n. with 2 µg of either adjuvant, no salivary IgA was detected (**Figure 2C**). These results further support that the 5 µg dose of adjuvants was superior to 2 µg dose in enhancing both systemic and mucosal responses. Therefore, we selected the 5 µg dose of both adjuvants for further experiments.

Taken together, i.n. immunization with Pn1-CRM_197_ and either dmLT or mmCT enhances induction and persistence of mucosal and systemic IgA responses.

### 3.3 dmLT and mmCT enhance early induction and homing of vaccine specific ASCs both after intranasal and subcutaneous immunization of neonates

**Figure 3:**
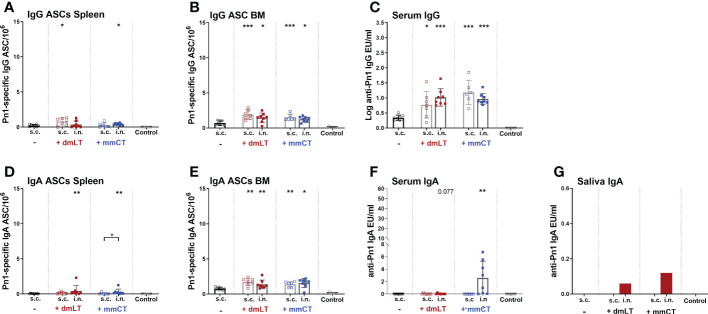
dmLT and mmCT both enhance early induction of mucosal and systemic humoral immune responses. IgG **(A–C)** and IgA **(D–G)** ASCs in spleen **(A, D)** and BM **(B, E)** and Abs in serum **(C, F)** and saliva **(G)** 14 days after immunization of neonatal mice with Pn1-CRM_197_ with or without adjuvants dmLT or mmCT s.c. or i.n. Results are expressed as number of spots/10^6^ cells (mean ± SD), IgG levels as (log mean EU/ml ± SD) or IgA levels (mean EU/ml ± SD) in 6–8 mice per group and statistical difference was calculated using Mann–Whitney U-test where adjuvant groups were compared to vaccine only group and immunization routes were compared between the adjuvanted groups. *p < 0.05, **p < 0.01,***p < 0.001.

Since we observed a difference in serum IgG and IgA levels between immunization routes we wanted to assess whether it could be explained by difference in the induction of ASCs in spleen, and their homing to BM early after immunization, at week 2. In particular, we wanted to assess whether the difference in serum IgG Abs, observed after week 4, between immunization routes could be explained by lower early induction of IgG^+^ ASCs after i.n. than s.c. immunization. To do so, neonatal mice were immunized i.n. or s.c. with Pn1-CRM_197_ with or without 5 µg of dmLT or mmCT, or injected with saline as a control. Spleen, BM, serum and saliva were collected 2 weeks after immunization (**Figures 3A–G**). At this early time point, mice immunized with either adjuvant had already enhanced numbers of Pn1-specific IgG^+^ and IgA^+^ ASC in BM, irrespective of the immunization route (**Figures 3B, E**). This was reflected in enhanced serum IgG Abs (**Figure 3C**) whereas in this experiment only mmCT enhanced serum IgA, following i.n. immunization (**Figure 3F**). Moreover, i.n. immunization with either adjuvant enhanced IgA^+^ ASC in the spleen compared to s.c. immunization with vaccine alone (**Figure 3D**) whereas IgG^+^ ASCs in spleen were somewhat increased, although only significantly for s.c. immunization with dmLT and i.n. immunization with mmCT (**Figure 3A**). Only the i.n. route induced salivary IgA Abs, 2 weeks after immunization with either adjuvant (**Figure 3G**), like previously observed at a later time point, or week 8 (**Figure 2C**). These results indicate that dmLT and mmCT may enhance the induction of vaccine-specific ASCs both after i.n and s.c. immunization of neonatal mice, although their effects are not as pronounced at this early time point. The numbers of IgG^+^ ASCs in spleen, homing to BM and IgG serum Abs were comparable between immunization routes, thus not explaining the difference in IgG serum Abs at later time points. Importantly, i.n. immunization is superior in inducing Pn1-specific IgA^+^ ASCs in spleen and IgA Abs in saliva.

### 3.4 dmLT and mmCT enhance neonatal germinal center induction irrespective of the immunization route

**Figure 4:**
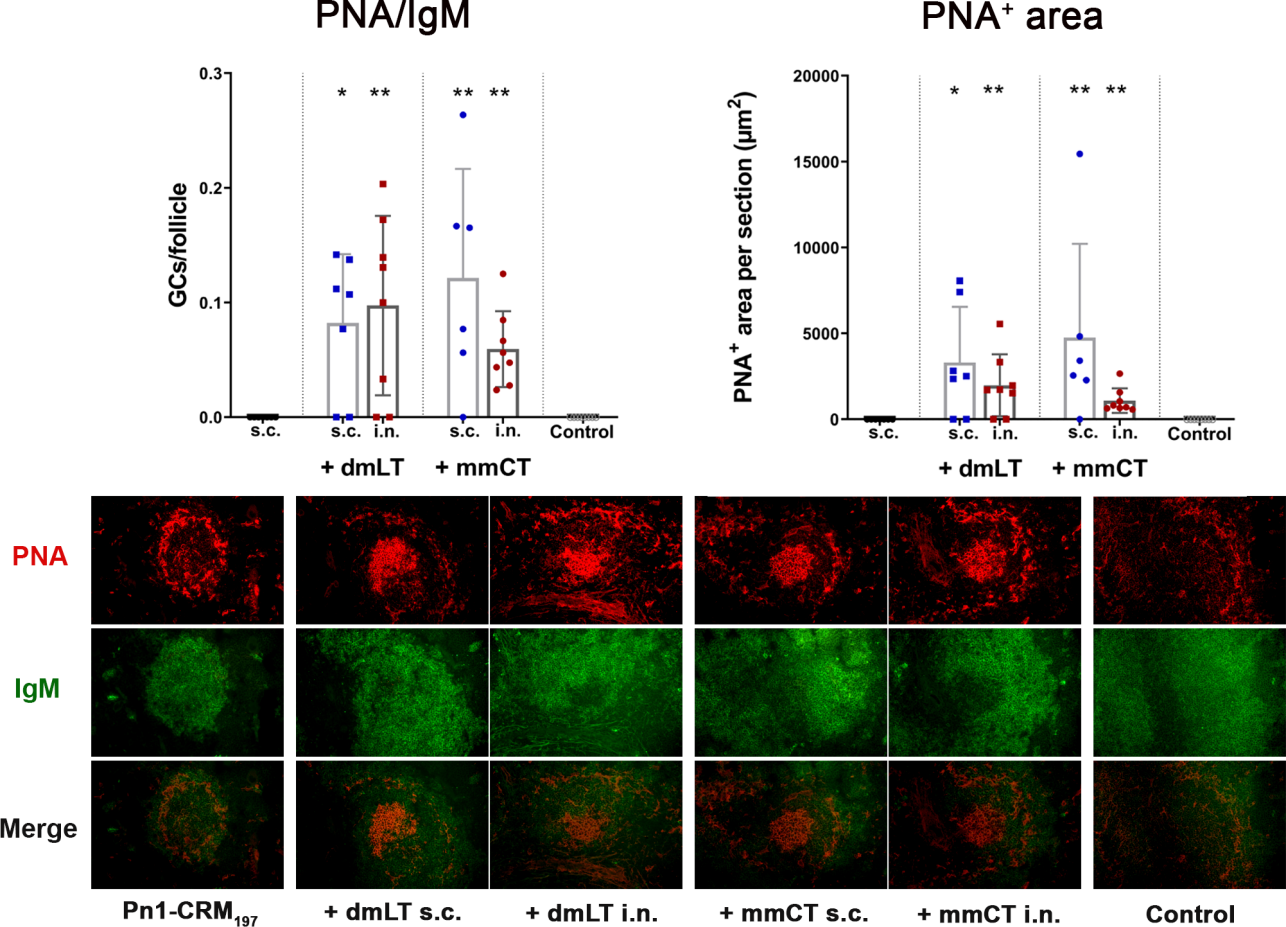
The adjuvants dmLT and mmCT enhance germinal center induction irrespective of the immunization route. Spleen sections were stained with anti-IgM and PNA 2 weeks after s.c. or i.n. immunization of neonatal mice with Pn1-CRM_197_ with/without 5 μg of dmLT or mmCT. PNA/IgM ratio represents activated GCs in relation to total number of follicles and PNA^+^ area represents total area of positive PNA staining per section. Results are expressed as mean ± SD in 6–8 mice per group and statistical difference was calculated using Mann–Whitney U-test where adjuvant groups were compared to vaccine only group and immunization routes were compared between the adjuvanted groups. *p < 0.05, **p < 0.01.

Since we observed no difference in numbers of IgG^+^ ASCs between routes early after immunization we wanted to assess whether the difference in IgG Abs after week 4 could be explained by difference in GC induction, as the GC reaction is fundamental for generation of long-lived Ab-mediated immunity (46) and it is one of the limiting factors in early life immune responses (5). We had stored fresh-frozen ½ of spleens from mice in the experiment described in chapter 3.3 and used those to stain for naive follicular B cells (IgM^+^) and GC B cells (PNA^+^) in spleen sections collected 2 weeks after immunization, the peak time for GC reaction in neonatal mice (5, 22). Both dmLT and mmCT enhanced GC formation in response to Pn1-CRM_197_ by both i.n. and s.c. immunization (**Figure 4**), demonstrating that the route of immunization does not affect the adjuvant-enhanced GC induction in spleen.

### 3.5 Intranasal immunization with dmLT or mmCT with higher doses of Pn1-CRM_197_ enhance both systemic and mucosal humoral responses

Mucosal delivery of vaccines faces several obstacles, including stability and dosage for optimal vaccine uptake at the mucosal inductive sites and since i.n. immunization with 0.75 µg of Pn1-CRM_197_ and 5 µg of dmLT or mmCT induced lower IgG Abs in serum than s.c. immunization, was suboptimal for reaching protective levels against bacteremia and induced more heterogeneous responses, we decided to assess i.n. immunization with higher doses of Pn1-CRM_197_ (1 µg, 2 µg and 4 µg) with 5 µg of dmLT or mmCT, and evaluated the immune response, by measuring Pn1-specific IgG and IgA Abs in serum bi-weekly, IgG^+^ and IgA^+^ ASCs in spleen and BM 8 weeks after immunization, as well as salivary IgA.

**Figure 5:**
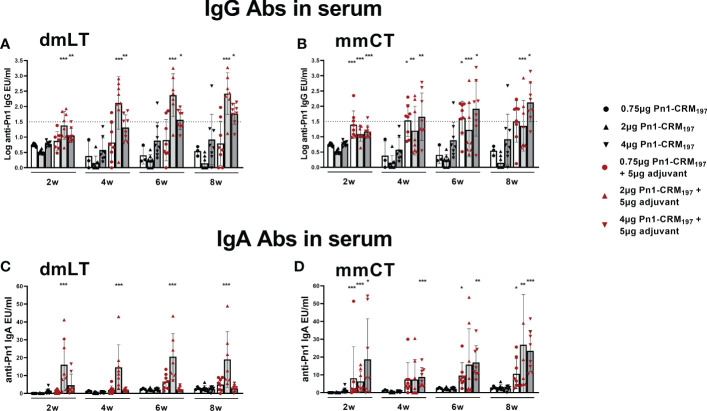
Increasing vaccine dosage *via* i.n. route enhances IgG Abs above protective levels for bacteremia. IgG **(A, B)** and IgA **(C, D)** Abs in serum 2, 4, 6 and 8 weeks after immunization of neonatal mice with different doses (0.75 µg, 2 µg, 4 µg) of Pn1-CRM_197_ alone s.c. or i.n. with dmLT or mmCT with comparable doses. Results are expressed as IgG levels (log mean EU/ml ± SD) or IgA levels (mean EU/ml ± SD) in 6–8 mice per group and statistical difference was calculated using Mann–Whitney U-test where adjuvant groups were compared to vaccine only group with the same dose of antigen. *p < 0.05, **p < 0.01, ***p < 0.001. The dotted line representes protective IgG Ab levels for pneumococcal bacteremia.

**Figure 6:**
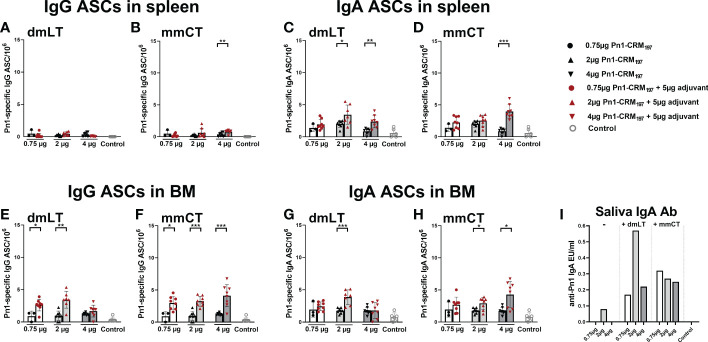
Effects of i.n. immunization with dmLT or mmCT on persistence of Pn1-specific AbSCs in spleen and bone marrow and Pn1-specific IgA Abs in saliva. IgG and IgA AbSCs in spleen **(A–D)** and BM **(E–H)**, and IgA in saliva **(I)** 8 weeks after immunization of neonatal mice with Pn1-CRM_197_ alone s.c. or with dmLT or mmCT intranasally. Results are expressed as number of spots/10^6^ cells (mean ± SD), IgG or IgA levels (EU/ml) in 6–8 mice per group and statistical difference was calculated using Mann–Whitney U-test where adjuvant groups were compared to vaccine only group. *p < 0.05, **p < 0.01, ***p < 0.001.

Pn1-specific IgG and IgA Ab levels in serum of neonatal mice immunized s.c. with 1, 2 or 4 µg of Pn1-CRM_197_ alone were low and comparable to those of saline-injected control mice (**Figure 5**). I.n. immunization with 2 µg of Pn1-CRM_197_ with dmLT or 4 µg Pn1-CRM_197_ with mmCT enhanced Pn1-specific IgG (**Figures 5A, B**) and IgA Abs (**Figures 5C, D**) at all time points compared to Pn1-CRM_197_ s.c. alone (p ≤ 0.001) and was comparable to s.c. immunization with mmCT or dmLT, respectively (**Supplementary Tables 5, 6**). Importantly, 86% (2µg Pn1-CRM_197_ + dmLT) and 88% (4µg Pn1-CRM_197_ + mmCT) of the mice reached protective IgG Ab levels against pneumococcal bacteremia. As previously observed, the inclusion of either adjuvant with Pn1-CRM_197_ enhanced the number of Pn1-specific IgG^+^ and IgA^+^ ASCs in BM (**Figures 6E–H**) whereas less effects were seen on ASCs in spleen as expected at this late time point (**Figures 6A–D**). Likewise, i.n. immunization with dmLT or mmCT enhanced salivary IgA Abs (**Figure 6I**) compared to s.c. immunization with vaccine alone. These results demonstrate that by increasing the dose of the vaccine when administered i.n. both dmLT and mmCT are able to enhance vaccine-specific IgG responses to comparable levels that of those reached by s.c. immunization with 0.75 µg Pn1-CRM_197_ and the adjuvants, in addition to the benefits achieved in mucosal and systemic IgA immune responses.

## 4 Discussion

The mucosae of the respiratory and gastro-intestinal system are the major entry ports for pathogens, and ideally, vaccines should be able to generate local protection against such infections. Neonatal vaccine development has been focused on injected vaccines and less on mucosal administration, with a few exceptions, like polio and rotavirus vaccines (47–49). However, there is extensive pre-clinical research on potential benefits of i.n. and oral vaccination against respiratory and gastrointestinal pathogens (40, 50–53). I.n. vaccination is an attractive strategy for defence against respiratory pathogens but requires adjuvants and/or special delivery systems to induce antigen-specific immunity, due to the presence of chemical and physical barriers such as digestive enzymes, ciliary movement, mucus and sneezing (15, 54).

Toxoid adjuvants CT and LT show strong adjuvanticity but also toxicity (55). Numerous strategies have been developed to enhance safety while retaining adjuvanticity (55, 56). Our group has shown that the adjuvant LT-K63 overcomes many limitations of the neonatal immune system and enhances immune responses (11, 12, 22, 25, 26, 57), but clinical development of LT-K63 was stopped due to transient adverse reactions in humans following i.n. delivery (58). Many studies have shown that dmLT and mmCT are strong, non-toxic adjuvants in mice (30–33). In this study, we assessed the potential of i.n. immunization of neonates with the adjuvants dmLT and mmCT, in particular if they can enhance induction of early life humoral immune responses leading to long-lasting protective immunity against pneumococcus. mmCT has been shown to enhance neonatal immune responses when given s.c. with a pneumococcal conjugate vaccine Pnc1-TT (25). Herein, we demonstrate that dmLT and mmCT enhanced both induction and persistence of neonatal immune response to another monovalent pneumoccocal conjugate vaccine, Pn1-CRM_197_, by both i.n. and s.c. immunization routes. As expected, i.n. immunization with dmLT and mmCT was generally superior in enhancing both local and systemic IgA responses, but a higher Pn1-CRM_197_ dose was needed for i.n. immunization to achieve protective IgG response comparable to that elicited by s.c. immunization with either adjuvant. The i.n. route requires lower doses of both adjuvant and antigen than oral immunization making it an attractive option for mucosal delivery of vaccines. However, following transient adverse reactions in clinical trials where LT-K63 was administered i.n. the field has generally steered away from nasal vaccination with GM1-bilding toxins such as LT-K63, dmLT and mmCT. Sublingual (s.l.) vaccination could also be an alternative to the i.n. route. It has been shown to induce both systemic and mucosal immune responses and importantly and contrary to nasal administration it does not seem to redirect antigens and/or adjuvants to the brain (59). Thus, comparison of systemic and mucosal response of neonates to i.n. and s.l. immunization with dmLT or mmCT is warranted. Importantly clinical trials showed that dmLT given orally to humans does not cause adverse reactions (38–40).

For both mmCT and dmLT we observed a stronger immune response with 5 µg than 2 µg of adjuvant by both routes after only one immunization. Additionally, no salivary IgA was detected after immunization with 2 µg of either adjuvant at either route, supporting that the 5 µg dose of the adjuvants is superior for enhancing both systemic and mucosal responses in neonates. Surprisingly, i.n. immunization with Pn1-CRM_197_ with dmLT or mmCT elicited lower Pn1-specific IgG levels than s.c. immunization, whereas in a previous study with Pnc1-TT and the adjuvant LT-K63, we found i.n. immunization to be superior to s.c. immunization in the induction of primary Pn1-specific IgG Ab responses in neonates with the same dose of the vaccine (11). Despite mmCT and dmLT being genetically detoxified CT and LT derivatives with similar structure as LT-K63, these are still different adjuvants. Herein, we observed more heterogeneous and generally lower responses when using dmLT than mmCT as adjuvants in vaccine formulations for neonatal mice. Other possible explanation could be that the CRM_197_ carrier may be less immunogenic than the TT carrier and PS-protein ratios may differ between the two conjugate vaccines and affect their immunogenicity. Even though s.c. immunization with 0.75 µg Pn1-CRM_197_ induced higher IgG levels than i.n. immunization this difference was only detected after week 4 following immunization with either adjuvant. This difference could not be explained by enhanced induction of IgG^+^ ASCs after s.c. immunization early after immunization since both routes elicited similar IgG levels and IgG^+^ ASCs in spleen and BM 2 weeks after immunization. Despite the difference in serum IgG Ab levels at later time points, i.n. and s.c. immunization with either adjuvant yielded comparable persistence of Pn1-specific IgG^+^ ASCs in BM 8 weeks after a single immunization, in agreement with earlier studies (60). IgG Abs accumulate in serum over time whereas the IgG^+^ ASCs assessment reflects their number at only the time point assessed in that particular lymphoid tissue. A plausible explanation for the difference in IgG Abs between the immunization routes at later time points could be that s.c. immunization induces higher numbers of short-lived ASCs, possibly in other lymphoid tissues not assessed in this study, like draining lymph nodes, that contribute to higher levels of accumulative IgG serum Abs over time.

Long-lived plasma cells persist in the BM, where they locate in survival niches possessing cellular and molecular factors that enable plasma cells to produce Abs over an individual’s lifespan (61). In this study we compared immunization routes with respect to induction and persistence of Pn1-specific ASCs in the spleen and BM. It is well established that long-lived BM IgG^+^ plasma cells contribute to protective Abs (22, 25, 26, 42), but is less clear for IgA^+^ plasma cells elicited by mucosal immune responses, as the majority of ASCs in the mucosa is rather short-lived (62, 63). However, there might be a long-lived compartment present in the mucosa as well (64–66), as is now generally accepted for the BM. It has been demonstrated both in mice and humans that plasma cells induced at mucosal sites were not only restricted to the mucosal tissue but also homed to the BM, where they produced systemic IgA (65, 67). In line with that, we found that the numbers of IgA^+^ ASC in BM were comparable 2 weeks after i.n. or s.c. immunization, whereas salivary IgA Abs were only detected in mice immunized i.n. Eight weeks after immunization, mice immunized i.n. with the adjuvants had significantly higher numbers of IgA^+^ ASC in BM than mice immunized s.c. and salivary IgA Abs were still high.

The effector functions of serum IgA Abs are not well defined. It has been reported that IgA can cross-link FcRαI expressed by myeloid cells such as monocytes, neutrophils, some subsets of macrophages and DCs, leading to secretion of cytokines and chemokines, degranulation, phagocytosis and formation of neutrophil extracellular traps (NETs) (68–72). Using humanized monoclonal Ab against meningococcal PorA, IgA (both IgA1 and IgA2) proved equally active to IgG (IgG3) in stimulating polymorphonuclear leukocyte (PMN) respiratory burst at physiological Ab concentrations (≤2 μg/ml). Furthermore, binding of monomeric, dimeric and polymeric serum IgA *via* FcαRI on PMN resulted in significant phagocytosis of heat-killed meningococci whereas secretory IgA was unable to activate phagocytosis (72). Thus, serum IgA is important for protection against bacteria by opsonophagocytosis and respiratory burst. As for respiratory viruses, elevated IgA serum levels have been shown to correlate with influenza vaccine efficacy (73, 74) and SARS-CoV-2 neutralization has been reported to correlate more closely with serum IgA than IgM or IgG during the first weeks after symptom onset (75). Herein, we observed that the effects of the adjuvants on serum IgA levels were most pronounced at early time points and like mentioned above, mice immunized i.n. with Pn1-CRM_197_ and 5µg of dmLT or mmCT had high salivary IgA levels both at early and later time points. These results are in agreement with higher salivary IgA after i.n. than s.c. immunization of neonatal, infant and adult mice with Pnc1-TT plus LT-K63 (11). Similar results were reported in adult mice immunized with different antigens and mmCT, where the adjuvant enhanced mucosal IgA Abs after i.n. and oral immunization (31, 33). Likewise, sublingual immunization of adult mice with an inactivated polio vaccine and dmLT enhanced both mucosal and serum IgA Abs, while mice immunized intramuscularly did not induce detectable IgA (35). Also in mice, i.n. vaccination with SARS-CoV proteins has been shown to induce both localized and systemic specific IgA responses that provided better protection against SARS-CoV challenge than intramuscular delivery, suggesting that mucosally induced IgA is important for protection against disease (76).

Although all immunized groups assessed here showed good protection against pneumococcal bacteremia, IgG levels were lower than needed for protection against pneumococcal lung infection, and there is a wide variation in immune responses after one immunization of neonatal mice, even when adjuvants are included in the vaccine formulation (11). The vaccination strategy might be optimized to achieve persistent highly protective vaccine-specific Ab levels, as observed for LT-K63 in neonatal mice (11, 12).

Taken together, we have shown that both dmLT and mmCT enhanced induction and persistence of the neonatal immune response to a pneumococcal conjugate vaccine, by only one i.n. or s.c. immunization. Both adjuvants given with Pn1-CRM_197_ by s.c. or i.n. routes similarly enhanced GC reaction, that are crucial for production of high affinity, class-switched Abs needed for protective immunity. However, the i.n. route was clearly superior to s.c. route for both induction and persistence of Pn1-specific salivary IgA, serum IgA and IgA^+^ ASCs in BM, although Pn1-specific IgG levels were lower at most timepoints, despite comparable numbers of IgG^+^ ASCs in BM 8 weeks after immunization. However, by increasing the i.n. dose of Pn1-CRM_197_ with either dmLT or mmCT, protective serum IgG levels could be induced to comparable levels to those elicited by the lower dose of vaccine and adjuvant by the s.c. route. Our study indicates that dmLT and mmCT are promising adjuvants for development of safe and effective mucosal and parenteral vaccination strategies against respiratory pathogens in early life.

## Data availability statement

The raw data supporting the conclusions of this article will be made available by the authors, without undue reservation.

## Ethics statement

The animal study was reviewed and approved by Experimental Animal Committee of Iceland (license number 2021-01-04), Austurvegi 64, Selfoss, Iceland. The study was carried out in accordance with Act No. 55/2013 on animal welfare and regulations 460/2017 on protection of animals used for scientific research.

## Author contributions

JM, AA, IJ, and SB conceived and designed the study, interpreted the results and wrote the manuscript. IJ and SB supervised the study. JM, AA, PF and SB performed the experiments. JM analyzed the data. All authors contributed to and approved the final version of the manuscript. All authors contributed to the article and approved the submitted version.
