# Supplementary material for: The adjuvants dmLT and mmCT enhance humoral immune responses to a pneumococcal conjugate vaccine after both parenteral or mucosal immunization of neonatal mice
Source: Front Immunol. 2023 Jan 20;13:1078904. doi: 10.3389/fimmu.2022.1078904 (PMC9896006; doi:10.3389/fimmu.2022.1078904)
Supplement: Supplementary file 1 [file DataSheet_1.docx]

Supplementary Material

The adjuvants dmLT and mmCT enhance humoral immune responses to a pneumococcal conjugate vaccine after both parenteral or mucosal immunization of neonatal mice

Jenny Lorena Molina Estupiñan ^1, 2^, Audur Anna Aradottir Pind^2^, Poorya Foroutan Pajoohian^1,2^, Ingileif Jonsdottir^1,2^, Stefania P. Bjarnarson^1,2*^

^1^Department of Immunology, Landspitali, The National University Hospital of Iceland, Reykjavik, Iceland.

^2^Faculty of Medicine, School of Health Sciences, University of Iceland, Reykjavik, Iceland.


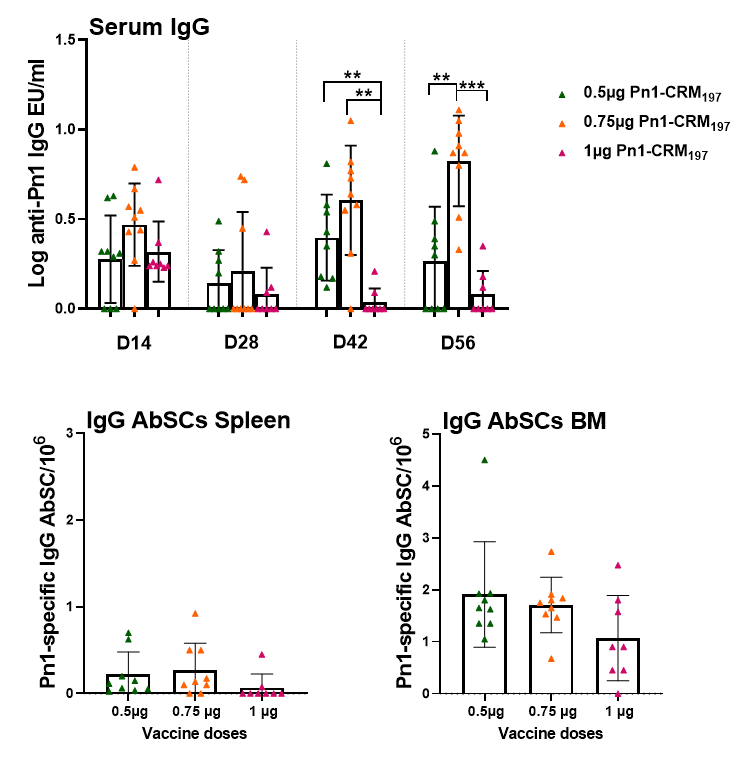


**Supplementary Figure 1.** Comparison of 3 doses of the pneumoccocal conjugate vaccine Pn1-CRM_197_ in neonatal mice. 7 day old mice were immunized subcutaneously (s.c.) with 0.5, 0.75 or 1 µg and measured Pn1-specific IgG antibodies bi-weekly upto 8 weeks. Results are expressed as number of spots/10^6^ cells ( mean ± SD), IgG levels (mean log EU/ml ± SD) in 8–9 mice per group and statistical difference was calculated using Mann–Whitney U-test.

**Supplementary Table 1.** **Pn1-specific IgG antibody responses 2, 4, 6 and 8 weeks after s.c. or i.n. immunization of neonatal mice with Pn1-CRM_197_ with or without 5 µg of adjuvants dmLT or mmCT.** Results are expressed as median values of log EU/mL ± SD. Statistical difference was calculated using Mann–Whitney U-test where adjuvant groups were compared to vaccine only group and immunization routes were compared between the adjuvanted groups, and p≤0.05, shown in red, was considered statistically significant.


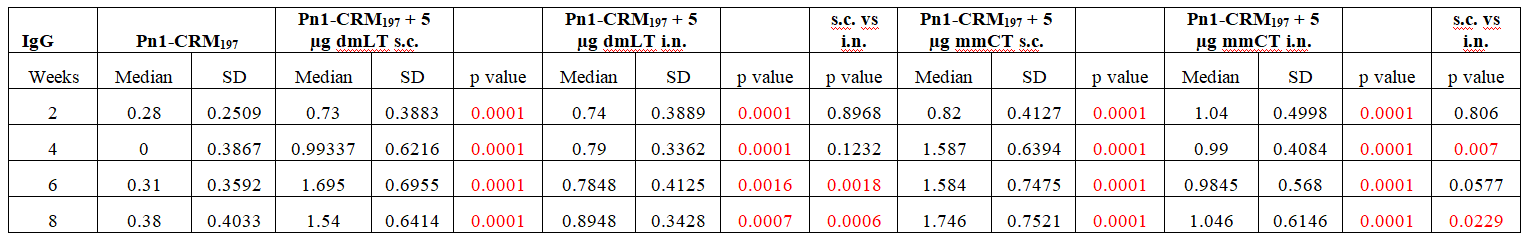


**Supplementary Table 2. Pn1-specific IgG antibody responses 2, 4, 6 and 8 weeks after s.c. or i.n. immunization of neonatal mice with Pn1-CRM_197_ with or without 2 µg of adjuvants dmLT or mmCT.** Results are expressed as median values of log EU/mL ± SD. Statistical difference was calculated using Mann–Whitney U-test where adjuvant groups were compared to vaccine only group and immunization routes were compared between the adjuvanted groups, and p≤0.05, shown in red, was considered statistically significant.


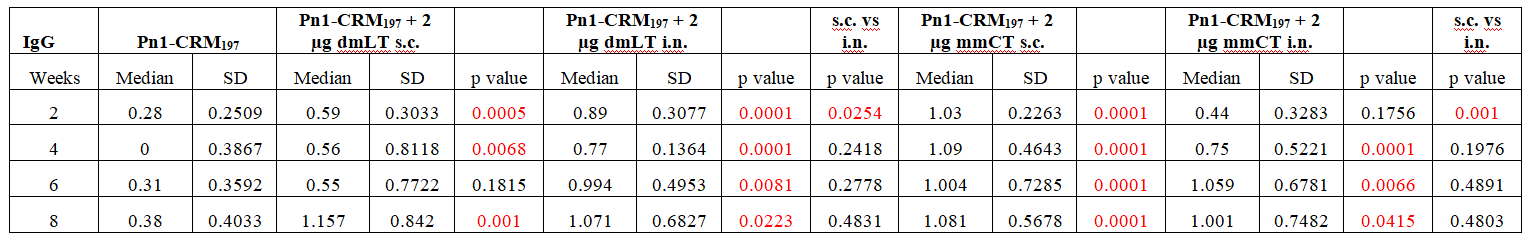


**Supplementary Table 3. Pn1-specific IgA antibody responses 2, 4, 6 and 8 weeks after s.c. or i.n. immunization of neonatal mice with Pn1-CRM_197_ with or without 5 µg of adjuvants dmLT or mmCT.** Results are expressed as median values of EU/mL ± SD. Statistical difference was calculated using Mann–Whitney U-test where adjuvant groups were compared to vaccine only group and immunization routes were compared between the adjuvanted groups, and p≤0.05, shown in red, was considered statistically significant.


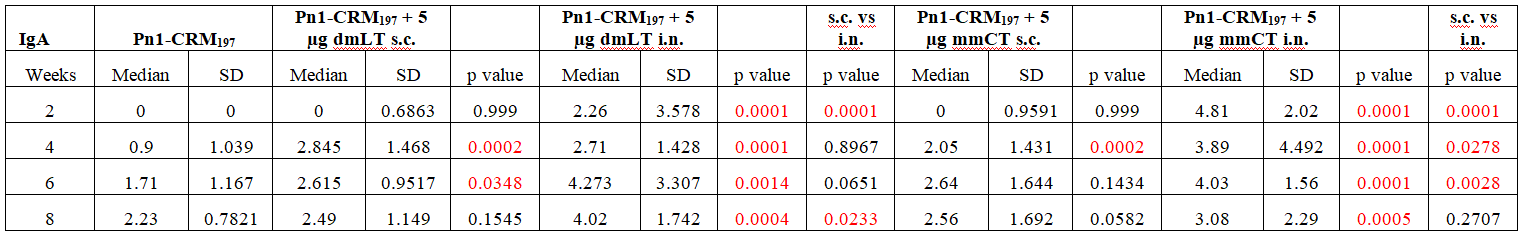


**Supplementary Table 4. Pn1-specific IgA antibody responses 2, 4, 6 and 8 weeks after s.c. or i.n. immunization of neonatal mice with Pn1-CRM_197_ with or without 2 µg of adjuvants dmLT or mmCT.** Results are expressed as median values of EU/mL ± SD. Statistical difference was calculated using Mann–Whitney U-test where adjuvant groups were compared to vaccine only group and immunization routes were compared between the adjuvanted groups, and p≤0.05, shown in red, was considered statistically significant.

**
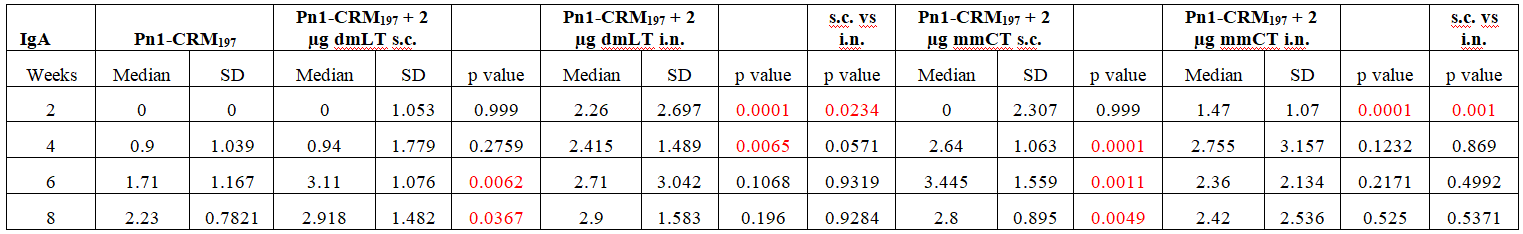
**

**Supplementary Table 5. Pn1-specific IgG antibody responses 2, 4, 6 and 8 weeks after immunization of neonatal mice with Pn1-CRM_197_ alone s.c. or with dmLT or mmCT intranasally.** Results are expressed as median values of log EU/mL ± SD. Statistical difference was calculated using Mann–Whitney U-test where adjuvant groups were compared to vaccine only group with the same dose of antigen, adjuvant groups were also compared to 0.75µg of the vaccine + adjuvant i.n. and s.c., and p≤0.05, shown in red, was considered statistically significant.

**
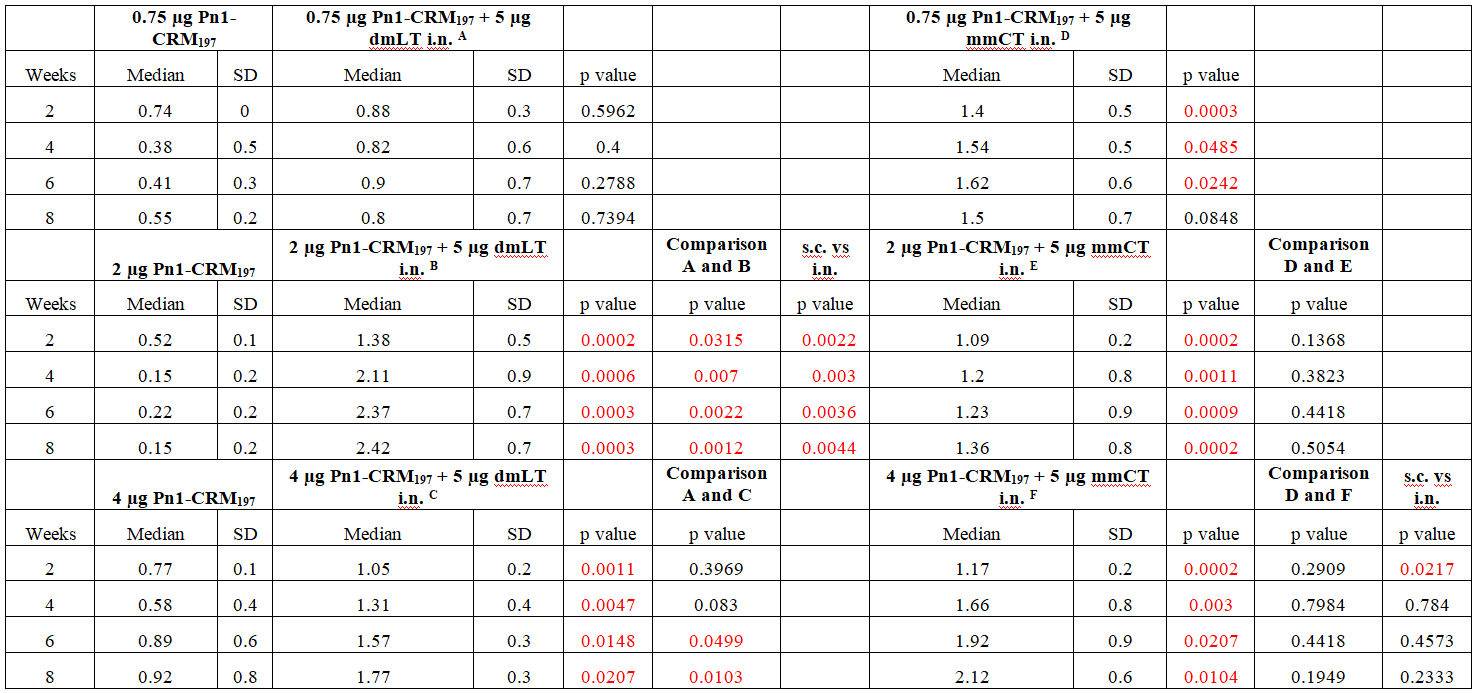
**

**Supplementary Table 6. Pn1-specific IgA antibody responses 2, 4, 6 and 8 weeks after immunization of neonatal mice with Pn1-CRM_197_ alone s.c. or with dmLT or mmCT intranasally.** Results are expressed as median values of log EU/mL ± SD. Statistical difference was calculated using Mann–Whitney U-test where adjuvant groups were compared to vaccine only group with the same dose of antigen, adjuvant groups were also compared to 0.75µg of the vaccine + adjuvant i.n. and s.c., and p≤0.05, shown in red, was considered statistically significant.

**
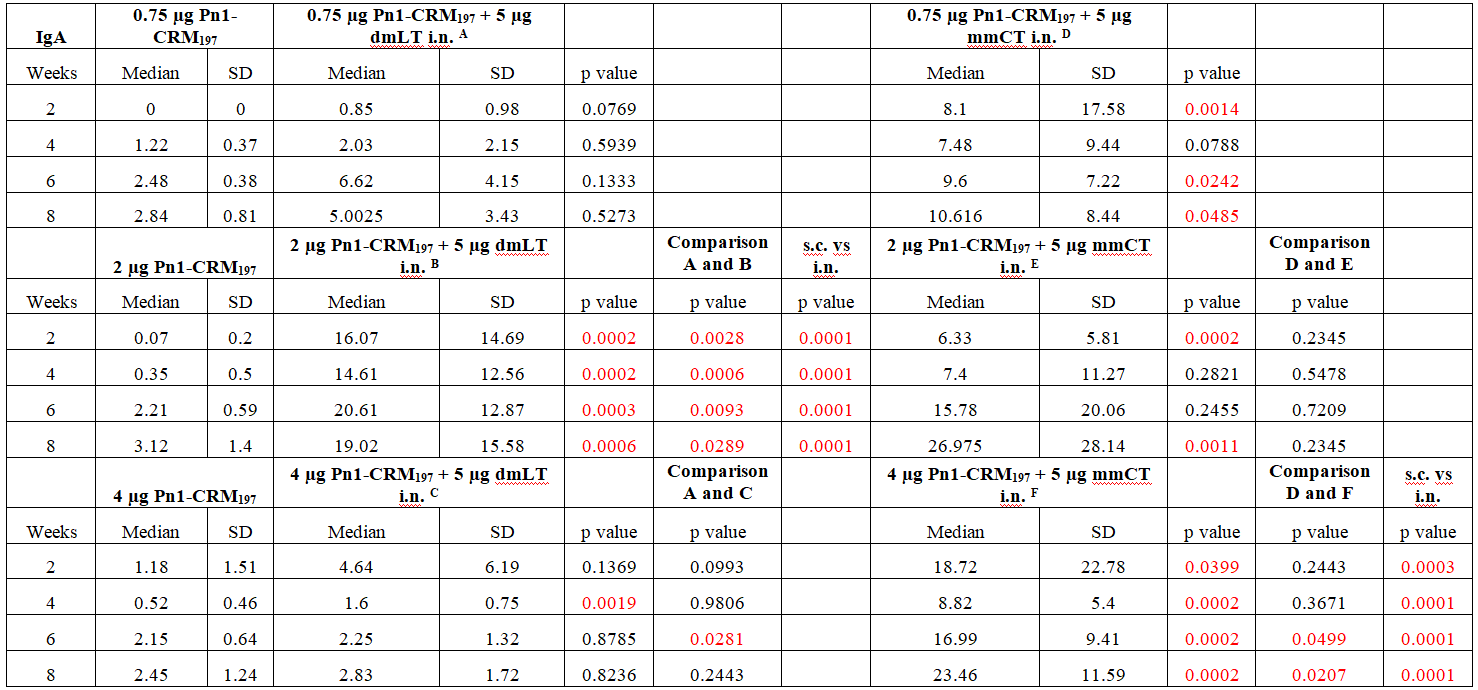
**
